# First Steps in Using Multi-Voxel Pattern Analysis to Disentangle Neural Processes Underlying Generalization of Spider Fear

**DOI:** 10.3389/fnhum.2016.00222

**Published:** 2016-05-27

**Authors:** Renée M. Visser, Pia Haver, Robert J. Zwitser, H. Steven Scholte, Merel Kindt

**Affiliations:** ^1^Department of Clinical Psychology, University of AmsterdamAmsterdam, Netherlands; ^2^Amsterdam Brain and Cognition, University of AmsterdamAmsterdam, Netherlands; ^3^Medical Research Council – Cognition and Brain Sciences UnitCambridge, UK; ^4^Department of Psychological Methods, University of AmsterdamAmsterdam, Netherlands; ^5^Department of Brain and Cognition, University of AmsterdamAmsterdam, Netherlands

**Keywords:** spider fear, multi-voxel pattern analysis (MVPA), fear generalization, fMRI, support vector machine, interpretation bias

## Abstract

A core symptom of anxiety disorders is the tendency to interpret ambiguous information as threatening. Using electroencephalography and blood oxygenation level dependent magnetic resonance imaging (BOLD-MRI), several studies have begun to elucidate brain processes involved in fear-related perceptual biases, but thus far mainly found evidence for general hypervigilance in high fearful individuals. Recently, multi-voxel pattern analysis (MVPA) has become popular for decoding cognitive states from distributed patterns of neural activation. Here, we used this technique to assess whether biased fear generalization, characteristic of clinical fear, is already present during the initial perception and categorization of a stimulus, or emerges during the subsequent interpretation of a stimulus. Individuals with low spider fear (*n* = 20) and high spider fear (*n* = 18) underwent functional MRI scanning while viewing series of schematic flowers morphing to spiders. In line with previous studies, individuals with high fear of spiders were behaviorally more likely to classify ambiguous morphs as spiders than individuals with low fear of spiders. Univariate analyses of BOLD-MRI data revealed stronger activation toward spider pictures in high fearful individuals compared to low fearful individuals in numerous areas. Yet, neither average activation, nor support vector machine classification (i.e., a form of MVPA) matched the behavioral results – i.e., a biased response toward ambiguous stimuli – in any of the regions of interest. This may point to limitations of the current design, and to challenges associated with classifying emotional and neutral stimuli in groups that differ in their judgment of emotionality. Improvements for future research are suggested.

## Introduction

The ability to recognize threatening stimuli clearly increases the chances of survival. Given that a known threat can take many forms, it is also adaptive to be cautious with other exemplars of the same semantic category that may predict a similar aversive outcome (Mineka, 1992). Stimulus generalization —a learning mechanism whereby conditioned responses extend to a range of stimuli resembling the original conditioned stimuli (Pavlov, 1927) – enables a fast response to novel potentially threatening stimuli. Yet, it can turn into maladaptive behavior when non-threatening stimuli or contexts are inappropriately treated as harmful. Here, we will refer to this phenomenon as ‘overgeneralization of fear’ or ‘maladaptive fear generalization.’ The way we use the term ‘fear’ here includes both physiological and behavioral responses to threat, as well as the subjective experience of fear (but see for an alternative use of the term ‘fear’ LeDoux, 2014).

Maladaptive fear generalization is a characteristic of anxiety disorders and post-traumatic stress disorder (Lissek et al., 2005, 2014; Mineka and Zinbarg, 2006; Kong et al., 2014; Bishop et al., 2015) and may even play a causal role in these disorders (Mathews and MacLeod, 2002; Wilson et al., 2006). For example, while in spider phobia fear responses to real spiders may be debilitating in itself, fear responses to stimuli that more or less resemble the object of fear (e.g., a piece of dust) may interfere most with daily functioning as phobic individuals find themselves in a permanent state of hypervigilance, avoiding many ‘safe’ situations (e.g., not eating tomatoes as their insides resemble the legs of a spider). Clarifying which processes enhance fear generalization will ultimately help to answer the fundamental question of why and how people differ in their disposition to develop maladaptive fears.

Based on decades of animal conditioning research that focused on the perceptual similarity and discriminability of threatening stimuli, it has been implicitly assumed that overgeneralization of fear is a perceptual deficit (Shepard, 1987). In line with this, examples from research in humans show that fearful individuals judge neutral faces as more negative (Richards et al., 2002; Bell et al., 2011), and that individuals with spider phobia more easily see a spider in pictures morphing from flowers to spiders (Kolassa et al., 2007). Only recently it became evident that fear generalization not solely depends on the physical properties of threatening stimuli but also on their conceptual properties (Dunsmoor et al., 2009, 2011, 2012; Soeter and Kindt, 2012, 2015; Kindt, 2014). This raises the question whether fear generalization observed for perceptual cues (such as when a piece of dust triggers a fear response) is in fact a perceptual process, or may instead emerge at a later stage of processing, when activated fear associations start guiding (biasing) the interpretation of a stimulus.

As the observed behavior does not reveal whether overgeneralization of fear already occurs during the initial perception and categorization of a stimulus, or emerges at a later stage, it is necessary to go beyond behavioral observations to study the (neural) processes that drive these behaviors. A number of studies have begun to elucidate brain processes involved in fear-related perceptual biases. These studies mainly found heightened sensory sensitivity to all external stimuli in high fearful individuals, expressed as enhanced early (100 ms) event-related potentials (ERPs; Kolassa et al., 2007, 2009; Frenkel and Bar-Haim, 2011; Weymar et al., 2013) and heightened responses in visual areas to phobogenic objects, often paralleled by heightened responses in the amygdala (Dilger et al., 2003; Straube et al., 2006; Alpers et al., 2009). These findings are in line with the commonly observed fear-related attentional bias (Bar-Haim et al., 2007), suggesting that fear facilitates afferent cortical processing in the human visual cortex when individuals search for potential threat. However, although heightened sensory sensitivity may explain a faster *detection* of a stimulus, it does not necessarily imply or explain a biased *classification* of that stimulus.

Studies on normal fear generalization have found that varying degrees of perceptual resemblance to a conditioned stimulus elicit graded responses (generalization curves) in the same neurocircuitry as is involved in the acquisition and expression of conditioned fear (i.e., insula, dorsal anterior cingulate cortex; Dymond et al., 2014), and in salience processing in general (e.g., the ventral tegmental area; Cha et al., 2014). While normally these graded responses inversely relate to activation in inhibitory brain systems, such as the hippocampus and the ventromedial prefrontal cortex, individuals with generalized anxiety seem specifically impaired in recruiting these systems, broadening the range of stimuli to which they respond with fear (Greenberg et al., 2013; Cha et al., 2014; Bishop et al., 2015).

Even though the aforementioned studies provided useful insights into the brain areas that are hyper- or hypoactive in anxiety disorders, they do not distinguish between a perceptual and a conceptual account of overgeneralization of fear in anxiety disorders. An increased neural response to a stimulus does not specify how that stimulus is categorized (e.g., high anxious individuals may exhibit heightened sensory sensitivity to virtually all stimuli, while only displaying a classification bias for a subset of stimuli). Univariate techniques therefore seem unsuited for addressing the question as to where in the cortical hierarchy ambiguous stimuli are originally marked as threatening.

In contrast, multi-voxel pattern analysis (MVPA) evaluates the information across groups of voxels, to characterize the unique neural representation of a stimulus within a certain brain region (Haxby et al., 2001). By training a classifier on neural patterns related to distinct stimulus classes one can classify patterns related to novel stimuli, providing a more sensitive way to assess the degree to which different stimuli or cognitive states are alike (Haynes and Rees, 2005; Kamitani and Tong, 2005; Norman et al., 2006; Kriegeskorte et al., 2008), or altered by fear (Li et al., 2008; Visser et al., 2011, 2013, 2015, 2016; Dunsmoor et al., 2014).

Here, we combined functional magnetic resonance imaging (fMRI) with an adapted version of the task used by Kolassa et al. (2007), to study overgeneralization of fear in individuals with low and high fear of spiders. Based on previous work, we predicted that individuals with high spider fear (HSF) would be more likely to classify ambiguous morphs as spiders than individuals with low spider fear (LSF). Furthermore, we examined in a data-driven manner whether overgeneralization of fear is associated with functional anomalies in regions traditionally associated with (1) early perception and object identification (Ungerleider and Haxby, 1994), which would support a perceptual account, and/or 2) regions involved in saliency (Etkin and Wager, 2007; Seeley et al., 2007; Hermans et al., 2011; Ipser et al., 2013), and higher cognitive processes (Miller and Cohen, 2001), which would support a more conceptual account. Of course, if a bias is already present in low-level areas it is likely to be present in higher-level areas as well, given that these areas respond to the information relayed from lower-level areas. If, however, a bias does not emerge until later in the processing stream, this could be an indication that the stimulus is initially correctly identified as non-threatening. Yet higher in the processing-stream certain features of the stimulus may still trigger semantic associations with the feared object, which in turn may evoke unpleasant feelings and biased decision making. Using support vector machine (SVM) classification in functional regions of interest we assessed at what point in the information-processing stream (i.e., ‘low-level’ visual areas, and/or ‘higher’ areas associated with the attribution of significance and decision making) this bias would become apparent.

## Materials and Methods

### Participants

Participants were recruited by means of advertisements in newspapers, social media and the university website. Selection was based on self-reported spider fear as measured by the Spider Phobic Questionnaire (SPQ; Klorman et al., 1974), with scores above 16 representing HSF and scores below 6 representing LSF. Of the 44 participants that were initially included, one participant was excluded because of excessive sleepiness, three participants because they did not comply with task instructions, and two participants because of excessive head motion. The final sample included 18 participants in the HSF condition (all females, three left-handed, mean = 24.1 ± 5.9 SD years of age), and 20 participants in the LSF condition (14 females, two left-handed, mean = 22.9 ± 1.8 SD years of age). Participants earned 20, – for their participation. All participants gave their written informed consent before participating and had normal or corrected-to-normal vision. None of the participants had knowledge of the Chinese language (see Materials and Methods). Procedures were executed in compliance with relevant laws and institutional guidelines, and were approved by the University of Amsterdam’s ethics committee (2014-CP-3390).

### Apparatus and Materials

#### Stimuli

**FIGURE 1:**
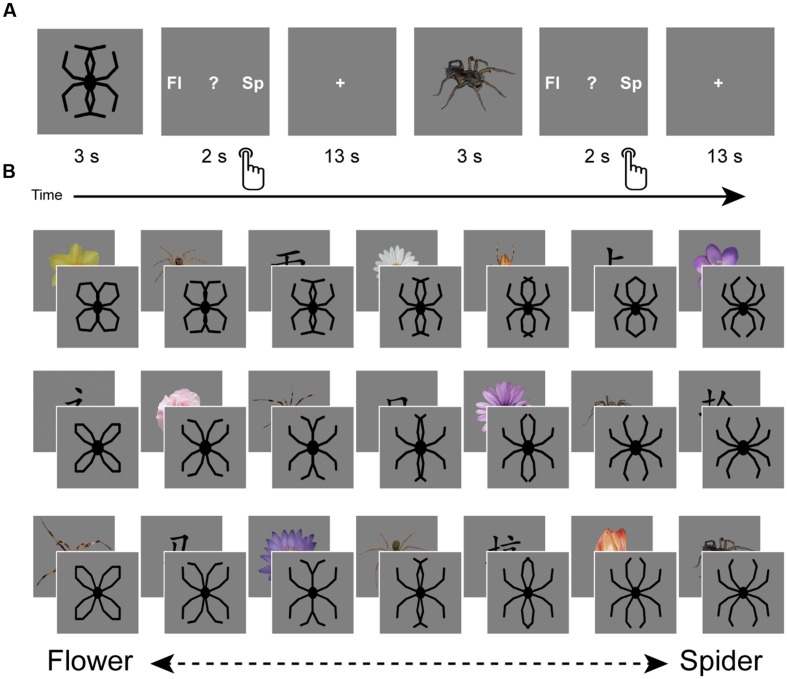
**Design.** The experiment consisted of one session of fMRI scanning during which a generalization task was performed **(A)**. This task consisted of the presentation of schematic flowers morphing to spiders (generously provided by Kolassa et al., 2007), intermitted by pictures of spiders, flowers and Chinese characters. Participants had to indicate whether they saw a spider (‘sp’), a flower (‘fl’) or none of the two (‘?’). Response buttons were counterbalanced over participants. Three variations existed of this flower-spider continuum **(B)**. Each variation was presented once, but the fixed order of stimulus presentation (counterbalanced over participants) was designed in such a way that priming effects could be averaged out: each step of the continuum was once preceded by a flower, once by a spider and once by a Chinese character. Images are not to scale.

The experiment consisted of one session of fMRI scanning, during which participants performed a task aimed to assess overgeneralization of spider fear (**Figure 1A**). This task was a modified version of the task used by Kolassa et al. (2007), who generously provided part of the stimulus material. This material consisted of schematic morphs that gradually transformed from a flower into a spider by shifting the outlines of the petals until they turned into spider legs (**Figure 1B**). Three variations existed of this continuum, with each continuum consisting of seven steps, yielding a total of 21 morphs. The presentation of a morph was alternated with the presentation of an unambiguous picture (**Figure 1B**), which was either a spider (*n* = 7), a flower (*n* = 7), or a Chinese character (*n* = 7). We collected these unambiguous pictures from the Web, adjusted their luminance, and separated them from their original background. Both the morphs and unambiguous pictures were presented on a gray background.

#### Subjective Measures

Fear of spiders was assessed with the SPQ (Klorman et al., 1974) and used to select participants. Prior to the experiment, trait anxiety and anxiety sensitivity were assessed with the Trait Anxiety inventory (STAI-T; Spielberger, 1983) and the Anxiety Sensitivity Index (ASI; Peterson and Reiss, 1993), respectively. State anxiety was assessed before and after the scanning procedure with the State Anxiety inventory (STAI-S; Spielberger, 1983).

#### Image Acquisition

Scanning was performed on a 3T Philips Achieva TX magnetic resonance imaging (MRI) scanner using a 32-channel head-coil. Functional data were acquired using a gradient-echo, echo-planar pulse sequence (TR = 2000 ms; TE = 27.63 ms; FA = 76.1°; 37 axial slices with ascending acquisition; 3 mm × 3 mm × 3.3 mm voxel size; 80 × 80 matrix; 240 × 133.98 × 240 FoV) and consisted of 415 dynamics. Foam pads minimized head motion, and online motion correction was applied by comparing each recorded volume to the initially recorded volume and adjusting the plane of recording with the displacement. A high-resolution 3D T1-weighted image (TR = 8.30 ms, TE = 3.82 ms, FA = 8°; 1 mm × 1 mm × 1 mm voxel size; 240 × 220 × 188 FoV) was additionally collected for anatomical visualization. Stimuli were backward-projected onto a screen that was viewed through a mirror attached to the head-coil.

#### Pre-processing

Functional magnetic resonance imaging data processing was carried out using FEAT (FMRI Expert Analysis Tool) Version 6.00, part of FSL (FMRIB’s Software Library^1^). Pre-processing included motion correction using MCFLIRT (Jenkinson et al., 2002); slice-timing correction; non-brain removal using BET (Smith, 2002); high-pass temporal filtering (*σ* = 50 s), 5 mm spatial filtering and pre-whitening (Woolrich et al., 2001). Functional images were coregistered to each individual’s high resolution structural images using FLIRT (Jenkinson and Smith, 2001; Jenkinson et al., 2002). Registration from high resolution structural to standard space (MNI152 template, 2 mm) was then carried out using FNIRT non-linear registration (Andersson et al., 2007).

### Experimental Design

Upon arrival participants were screened and instructed about the scanning procedure. The experiment started with a structural scan. During functional scanning participants performed the generalization task, viewing morphs (ambiguous) as well as pictures (unambiguous; **Figures 1A,B**). Participants were requested to make a response after each stimulus by pressing a button, indicating whether they had seen a spider, a flower, or none of the two (represented by a question mark). With regard to the morphs, we informed participants that the ‘drawings’ they would be seeing would resemble spiders or flowers to a certain degree, while a proportion of these drawings would resemble none of the two. We emphasized that responses to these drawings were purely subjective and that there were no right or wrong answers. Furthermore, we explicitly instructed participants to wait until the stimulus (3 s) disappeared and the response screen (2 s) was presented. Consequently, reaction times cannot be reliably interpreted, as they do not reflect the initial response to the picture. The response screen (**Figure 1A**) reminded participants which button to press (left, middle, or right), but only the first two letters of the options were shown (‘fl,’ ‘?,’ ‘sp’). The Chinese characters were included to introduce a clear ‘none-of-the-two’ category, so that responses to the morphs were not biased by response frequencies to the unambiguous stimuli. Stimulus presentation was fixed and was designed in such a way that priming effects could be averaged out: each step of the continuum was once preceded by a flower, once by a spider and once by a Chinese character (**Figure 1B**). Response buttons and stimulus presentation were counterbalanced across participants. Inter-trial intervals were fixed and relatively long (13 s), which seems optimal for single-trial pattern analysis (Visser et al., 2016). The task started with three practice trials (unambiguous pictures of a flower and a spider, and a Chinese character), which were discarded from further analysis. Participants were instructed to pay close attention to the pictures, even if pictures were unpleasant. Continuous eyetracker-recordings ensured that participants complied with these instructions.

### Univariate fMRI Analysis

In order to create functional regions of interest (ROIs), and to facilitate interpretation of the results in light of previous fMRI studies on spider fear, we ran a standard voxelwise whole-brain analysis, modeling all trials within a condition as one regressor [10 regressors in total: seven morph steps (three per step), unambiguous flowers (7), unambiguous spiders (7) and Chinese characters (7)] and including six motion parameters and temporal derivatives as regressors of no interest. Higher-level mixed-effects analyses were conducted to assess group differences in the contrast of interest, that is, unambiguous spiders > unambiguous flowers. Furthermore, we explored whether there were voxels which’ tuning curve followed the gradient of flowers morphing to spiders (i.e., we set up a contrast to test whether there was a linear increase or decrease as function of morphing) and whether these voxels showed overlap with the ones identified using the multivariate approach. Activation was thresholded at *Z* > 2.3 (*Z* > 3.1 for creating the ROIs) and cluster-corrected at *p* < 0.05. Finally, we plotted for each of the functional ROIs the average activation per stimulus type, to examine if the generalization curves mirrored the behavioral data.

### Region of Interest Selection

The parametric map obtained for the contrast unambiguous spiders > unambiguous flowers was thresholded at *Z* > 3.1 and masked with a whole brain mask created from the Harvard-Oxford cortical and subcortical atlas (part of the FSL software), which excluded brain stem and cerebellum and was thresholded at a probability of >10%. Next, 12 ROIs were created from clusters consisting of at least 100 adjacent voxels. Using these functional ROIs, we then classified the ambiguous stimuli using a SVM (see first two paragraphs of Section “Multi-voxel Pattern Analysis”). This selection of ROIs represented a sample of different areas in the information-processing stream, with a high likelihood of being responsive to the task (as areas that distinguish between unambiguous stimuli may also be responsive to gradual changes in their features).

### Multi-Voxel Pattern Analysis

Each trial was modeled as a separate regressor in a general linear model (GLM), including six motion parameters as regressors of no interest. The resulting parameter estimates were normalized to down-weight noisy voxels, by dividing each voxel’s parameter estimate by the standard error of that voxel’s residual error term after fitting the first-level GLM. In Matlab (version 8.0; MathWorks) we created for each participant, for each ROI a vector containing the normalized parameter estimates per voxel for a particular trial. Next, these vectors were used for classification analysis (next paragraph).

For each participant, we performed a leave-two-out classification analysis with 1,000 iterations within each functional ROI, using a two-class SVM with a linear Kernel function (LIBSVM, Chang and Lin, 2011), Software available at http://www.csie.ntu.edu.tw/ cjlin/libsvm. With each iteration two unambiguous stimuli (one flower and one spider) were separated as test set, while the other unambiguous stimuli (six flowers and six spiders) were used to train the classifier (random selection with replacement). Next, the two separated stimuli as well as the 21 ambiguous stimuli were classified, yielding a total number of 23 classifications per iteration (one flower, one spider, and 21 morphs). These classifications were averaged over iterations and over the different stimulus types (spider, flower and seven steps of the flower-spider continuum).

### Statistical Analyses

#### Behavioral Data

The behavioral responses, denoted by *Y*, were dichotomized (*Y* = 1 for “spider,” *Y* = 0 for other responses). To account for the nesting of stimulus responses within persons, the data were modeled with a mixed logistic regression model: *P*(*Y* = 1) = exp(*Z*)/(1+exp(*Z*)), where *P*(*Y* = 1) denotes the probability that *Y* = 1, and where *Z* denotes a linear combination of fixed and random effects. Note that these models could also be considered as mixed Rasch models (Rijmen et al., 2003; Kolassa et al., 2007). We considered four models: model 1 consisted of a fixed group effect (LSF vs. HSF, coded as 0 and 1, respectively) and a random person effect. Model 2 consisted of a fixed stimulus effect (the degree to which a stimulus resembles a spider, seven levels) and a random person effect. Model 3 consisted of a fixed stimulus effect, a fixed group effect, and a random person effect. Model 4 consisted of a fixed stimulus effect, a fixed group effect, a multivariate random person effect with a variance parameter for each group, and a covariance parameter between the two groups.

The model fit was evaluated with AIC and BIC fit statistics, as well as with likelihood ratio tests for nested models. The models were estimated with the statistical software package R, version 3.1.1. (R Core Team, 2014) using the glmer() function within the R-package ‘lme4’ (De Boeck et al., 2011; Bates et al., 2013).

#### SVM Classification

By iterating training and test sets we obtained normally distributed classification scores for the brain data. Hence, we used parametric tests to assess whether the classification of blood oxygenation level dependent (BOLD-MRI) patterns, obtained on the individual level, revealed on average more spider classifications in the HSF group, compared to the LSF group. Statistical tests were performed using SPSS (version 21). We first assessed per ROI, per group, whether the classification of the unambiguous spider and flower stimuli was above chance level (0.5), using a one-sample *t*-test, to get a sense of the reliability of the SVM classification in that area. Next, we performed a mixed between-within-subjects analysis of variance (ANOVA) within each ROI, with morph as within-subject factor and group as between-subject factor. We specifically tested whether there was a main effect of stimulus type (indicating that the region was sensitive to changes in stimulus features), and if significant or trend-significant, whether there was a main effect of group. Predictions were tested while correcting for multiple comparisons (the number of ROIs) by limiting the false discovery rate (Benjamini and Hochberg, 1995). In case that the assumption of sphericity was violated a Greenhouse–Geisser correction was applied. All *p*-values are reported two-sided, with the significance level set at α = 0.05.

## Results

### Participant Characteristics

Participants in the LSF and HSF group did not differ in trait anxiety and anxiety sensitivity (*F*_1,36_ = 0.04; *p* = 0.847; *F*_1,36_ = 1.22; *p* = 0.277, respectively; **Table 1**). In the HSF group, the (anticipated) confrontation with spider-related material was associated with higher state anxiety before scanning (*F*_1,35_ = 6.02, *p* = 0.019, $\eta_{p}^{2}$ = 0.15), and marginally higher state anxiety after scanning (*F*_1,35_ = 3.97, *p* = 0.054, $\eta_{p}^{2}$ = 0.10).

**Table 1:** Mean values ± SD of self-reported fear of spiders (SPQ), anxiety sensitivity (ASI), state anxiety (STAI-S, pre- and post-scan), and trait anxiety (STAI-T) per group.

	HSF (*n* = 18)	LSF (*n* = 20)
SPQ	21.0 (±3.0)^∗^	3.2 (±1.6)^∗^
ASI	8.3 (±4.2)	10.0 (±5.0)
STAI-T	35.2 (±9.5)	35.8 (±9.0)
STAI-S pre	36.4 (±9.1)^∗^	29.3 (±8.5)^∗a^
STAI-S post	35.7 (±13.8)^#^	28.5 (±7.4)^#a^


^a^Based on 19 participants. SPQ, spider phobia questionnaire; ASI, Anxiety Sensitivity Index; HSF, high spider fear; LSF, low spider fear. Significant and trend-significant group effects are marked as follows: *p < 0.050; ^#^*p* < 0.080.

### Behavioral Responses

**FIGURE 2:**
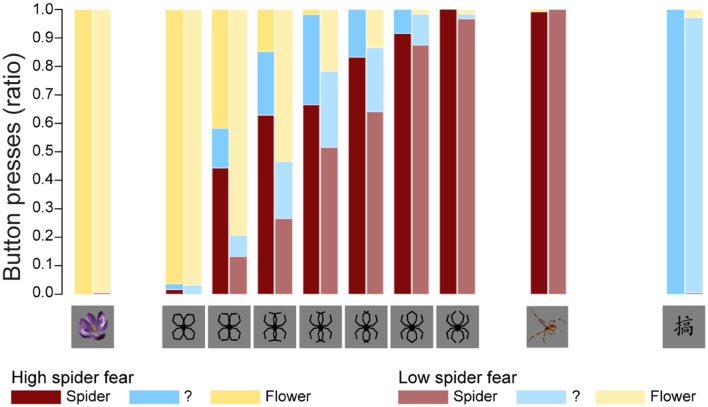
**Behavioral data.** The average proportions in both groups of stimuli identified as spiders, flowers, or neither/nor. These results replicate previous findings (Kolassa et al., 2007), suggesting that an individual with high fear of spiders is more likely to classify an ambiguous stimulus as a spider.

**Figure 2** displays the average proportions in both groups of stimuli identified as spiders, flowers, or neither/nor. **Table 2** summarizes the fit of the four models as described in the Section “Statistical Analyses.”

**Table 2:** Number of estimated parameters (df), log-likelihood (logLik), AIC, BIC, and Akaike weights of the four models described in section on Univariate fMRI Analysis.

	df	logLik	AIC	BIC	Akaike weights
Model 1	3	-529.72	1065.44	1079.48	0.000
Model 2	8	-303.43	622.86	660.32	0.042
Model 3	9	-299.53	617.05	659.19	0.771
Model 4	11	-298.95	619.89	671.40	0.186


The fit statistics indicate that model 3 (i.e., the model with a fixed stimulus effect, a fixed group effect, and a random person effect) was the best fitting model. This conclusion was supported by the results of the likelihood ratio tests in **Table 3**. The fit of model 3 was significantly better than the fit of models 1 and 2, while the fit of model 4 was not significantly better than the fit of model 3.

**Table 3:** Results likelihood ratio tests.

	-2log(Lik-ratio)	df	*p*
Model 1 vs. model 3	460.38	6	<0.0001
Model 2 vs. model 3	7.80	1	0.005
Model 3 vs. model 4	1.16	2	0.56


The parameter estimates of model 3 are displayed in **Table 4**. The interpretation of the parameters is as follows: since LSF is arbitrarily chosen as reference group, the probability that a randomly selected person with LSF will classify morph 3 as “spider” is exp(-0.9872)/(1+exp(-0.9872)) = 0.271, while the probability that a randomly selected person with HSF will classify morph 3 as “spider” is exp(-0.9872+1.4036)/(1+exp(-0.9872+1.4036)) = 0.603. Note that the estimates of the stimulus parameters increase from morph 1 to 7, which implies that the probability that a stimulus will be classified as spider increases from morph 1 to 7. The estimated group effect is statistically significant, *z* = 2.889, *p* = 0.004. These results replicate previous findings (Kolassa et al., 2007), suggesting that an individual with high fear of spiders is more likely to classify an ambiguous stimulus as a spider.

**Table 4:** Parameter estimates of model 3.

Parameter^a^	Estimate	*SE*
**Fixed effects:**		
Morph 1	-6.4112	1.1208
Morph 2	-2.0682	0.4191
Morph 3	-0.9872	0.3895
Morph 4	-0.2625	0.3827
Morph 5	0.6497	0.3896
Morph 6	1.9926	0.4402
Morph 7	3.7894	0.6796
Group	1.4036	0.4859
**Random effect:**		
Intercept person (*SD*)	1.314	


*^a^Estimates based on the following parameterization: glmer [Y ∼-1 + stimulus + group + (1 | person), family = binomial].*

### Univariate fMRI Results

**FIGURE 3:**
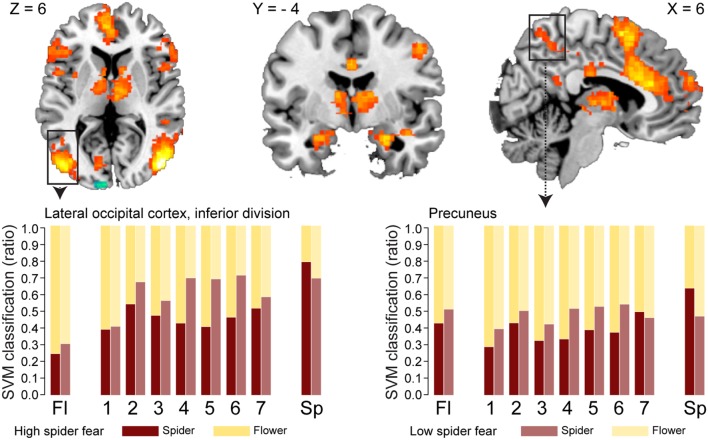
**Neural classification.** Univariate parametric maps showing clusters of voxels that discriminate between unambiguous flower and spider pictures (top panels, *Z* > 3.1, cluster corrected at *p* < 0.05). Bottom panels show the average SVM classifications in two functional ROIs. In the lateral occipital cortex response patterns were more often classified as spider in individuals with low spider fear (*p* < 0.029; uncorrected).

**FIGURE 4:**
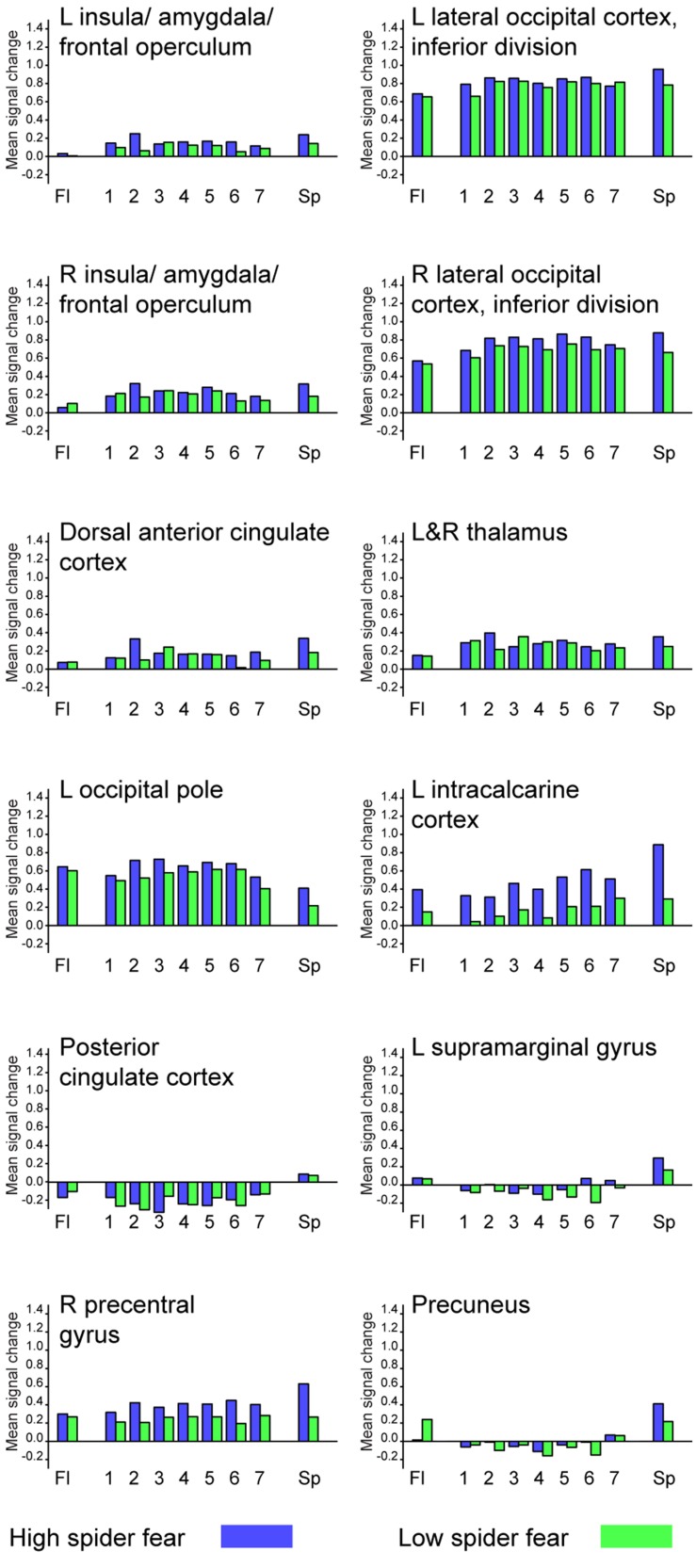
**Average activation per stimulus type, in 12 functional ROIs.** L = left; R = right.

Whole brain univariate analyses showed typical salience-network activation in response to spider pictures compared to flower pictures (**Table 5** and **Figure 3**). This effect was strongest in individuals with HSF, who showed more activation in the salience network as well as visual (association) areas, than individuals with LSF (**Table 5**). These results are in line with previous findings (Dilger et al., 2003; Straube et al., 2006; Alpers et al., 2009). Univariate analysis on activity related to the ambiguous stimuli revealed a cluster in the occipital cortex that responded more to ‘spiderness’ and a cluster in the dorsal paracingulate cortex that responded more to ‘flowerness,’ but no group differences were observed here (**Table 6**). Finally, **Figure 4** shows the average activation per stimulus type, in 12 functional ROIs. Although individuals with high fear of spiders showed on average more activation than individuals with low fear of spiders in the occipital cortex and a cluster comprising the left amygdala and insula, these effects only reached trend significance (*F*_1,36_ = 3.13; *p* = 0.085 and *F*_1,36_ = 3.20; *p* = 0.082, respectively) and were not specific for ambiguous stimuli (i.e., no effects of morph step in any of the ROIs).

**Table 5:** Brain areas showing differential activation for the unambiguous pictures (*n* = 38).

Brain region (COG)	MNI coordinates			Volume	
		
	*x*	*y*	*z*	# voxels	Maximum Z
Spider > Flower					

Group mean (*n* = 38)					
Salience network (i.e., frontoinsular cortex, orbitofrontal cortex, dorsal anterior cingulate extending into posterior cingulate cortex, paracingulate cortex, superior frontal gyrus and juxtapositional lobule cortex, temporoparietal junction, amygdala, thalamus, brainstem, cerebellum) and lateral occipital cortex	1	1	-27	69638	8.49
High spider fear (*n* = 18) > Low spider fear (*n* = 20)					
Lingual gyrus, precuneus, intracalcarine cortex	-3	-62	10	14658	4.91
R superior, middle frontal gyrus, precentral gyrus	23	4	54	1354	4.16
R insula, frontal operculum, orbitofrontal cortex	49	16	-5	1308	4.65
R middle temporal gyrus (temporooccipital part), lateral occipital cortex (inferior division), angular gyrus, parietal operculum cortex, supramarginal gyrus	56	-52	12	1137	4.13
Anterior cingulate cortex	1	18	25	1102	3.95
L insula, frontal operculum, orbitofrontal cortex	-39	12	-8	704	3.89
L parietal operculum cortex, supramarginal gyrus	-56	-39	28	520	3.8

Flower > Spider					

Group mean (*n* = 38)					
R precentral gyrus, R post-central gyrus	9	-31	67	473	3.60
High spider fear (*n* = 18) > Low spider fear (*n* = 20)					
No significant clusters					


*Whole brain activation (*Z* > 2.3, cluster-corrected at *p* < 0.05), showing clusters of voxels that discriminate between unambiguous spider and flower pictures, and within this contrast, activation that discriminates between groups. Coordinates are in MNI-space and depict for each significant cluster the Center of Gravity (COG). Labels are derived from the Harvard–Oxford cortical and subcortical atlases. L = left; R = right.*

**Table 6:** Brain areas showing differential activation for the ambiguous pictures (*n* = 38).

Brain region (COG)	MNI coordinates			Volume	
		
	*x*	*y*	*z*	# voxels	Maximum Z
More spider					

Group mean (*n* = 38)					
Occipital cortex	2	-80	8	8591	4.85
High spider fear (*n* = 18) > Low spider fear (*n* = 20)					
No significant clusters					

More flower					

Group mean (*n* = 38)					
Paracingulate gyrus, superior frontal gyrus	4	35	31	1633	3.64
High spider fear (*n* = 18) > Low spider fear (*n* = 20)					
No significant clusters					


*Whole brain activation (*Z* > 2.3, cluster-corrected at *p* < 0.05), showing clusters of voxels which’ tuning curves followed the gradient of flowers morphing to spiders and vice versa, and within these contrasts, activation that discriminates between groups. Coordinates are in MNI-space and depict for each significant cluster the Center of Gravity (COG). Labels are derived from the Harvard–Oxford cortical and subcortical atlases.*

### Classification Results

**Table 7** displays the proportions of correctly classified neural response patterns related to the unambiguous stimuli, in 12 functional ROIs. In most occipital areas, classification accuracy was significantly above chance for both the HSF and the LSF group. Additionally, in the HSF group classification was above chance in amygdala, insula, anterior cingulate cortex, supramarginal gyrus, and the precentral gyrus. An exploratory analysis showed that in these areas (ROI 3-5, 11-12) the classification of unambiguous spiders was significantly higher in the HSF group than in the LSF group (all *p*s < 0.018), suggesting that the pictures of real spiders elicited more generalized responses when individuals were afraid of spiders (Watts and Dalgleish, 1991).

**Table 7:** Mean values ± SD from the support vector machine classification of neural patterns related to presentations of unambiguous stimuli, in 12 functional ROIs.

Brain region	# voxels	Proportion of correctly classified flower stimuli		Proportion of correctly classified flower stimuli	
			
		HSF (*n* = 18)	LSF (*n* = 20)	HSF (*n* = 18)	LSF (*n* = 20)
ROI 1: L amygdala, anterior insula, inferior temporal gyrus, anterior division, inferior frontal gyrus, frontal operculum, frontal pole	3110	0.62^∗^ (± 0.22)	0.58^#^ (± 0.20)	0.70^∗^ (± 0.19)	0.62^∗^ (± 0.19)
ROI 2: L lateral occipital fusiform cortex, lateral occipital cortex inferior division, occipital fusiform gyrus	4542	0.76^∗^ (± 0.17)	0.69^∗^ (± 0.20)	0.79^∗^ (± 0.23)	0.69^∗^ (± 0.18)
ROI 3: R amygdala, anterior insula, inferior temporal gyrus, anterior division, inferior frontal gyrus, frontal operculum, frontal pole	3090	0.68^∗^ (± 0.18)	0.57 (± 0.18)	0.75^∗^ (± 0.17)	0.61^∗^ (± 0.16)
ROI 4: R lateral occipital fusiform cortex, lateral occipital cortex inferior division, occipital fusiform gyrus	4806	0.76^∗^ (± 0.14)	0.71^∗^ (± 0.14)	0.84^∗^ (± 0.16)	0.69^∗^ (± 0.11)
ROI 5: dorsal anterior cingulate cortex, paracingulate cortex, superior frontal gyrus, juxtapositional lobule cortex	5044	0.62^∗^ (± 0.18)	0.49 (± 0.17)	0.71^∗^ (± 0.15)	0.57 (± 0.18)
ROI 6: L and R thalamus	1125	0.57^#^ (± 0.16)	0.53 (± 0.14)	0.59^∗^ (± 0.17)	0.57^∗^ (± 0.15)
ROI 7: L occipital pole	113	0.61^∗^ (± 0.21)	0.64^∗^ (± 0.16)	0.68^∗^ (± 0.14)	0.63^∗^ (± 0.19)
ROI 8: L intracalcarine cortex	133	0.69^∗^ (± 0.17)	0.56 (± 0.18)	0.68^∗^ (± 0.18)	0.61^∗^ (± 0.19)
ROI 9: posterior cingulate cortex	122	0.56 (± 0.16)	0.58^∗^ (± 0.15)	0.57^#^ (± 0.14)	0.56 (± 0.16)
ROI 10: L supramarginal gyrus	397	0.60^#^ (± 0.23)	0.55 (± 0.14)	0.61^∗^ (± 0.21)	0.57^#^ (± 0.17)
ROI 11: R precentral gyrus	215	0.64^∗^ (± 0.15)	0.47 (± 0.14)	0.71^∗^ (± 0.17)	0.48 (± 0.15)
ROI 12: precuneus	301	0.57 (± 0.23)	0.49 (± 0.14)	0.63^∗^ (± 0.18)	0.47 (± 0.18)


One-sample t-tests were used to test if the proportion of correctly classified stimuli was significantly above chance level (0.5), with 17 degrees of freedom for the high spider fear (HSF) group and 19 degrees of freedom for the low spider fear (LSF) group. *p < 0.050; ^#^*p* < 0.080. L = left; R = right.

With regard to the morphs (ambiguous stimuli), no group differences were observed in the proportion of response patterns classified as spider (**Table 8**), except for a small (uncorrected) effect in the left lateral occipital cortex. This effects was even in the opposite direction as hypothesized, given that response patterns were more likely to be classified as spiders when individuals were not afraid of spiders (**Figure 3**, left panel).

**Table 8:** Summary of statistics of the support vector machine classification of neural patterns related to presentation of morphs (*n* = 38), in 12 functional ROIs.

Brain region	# voxels	Main effect of morph (7) (within-subject)			Main effect of group (2) (between-subject)		
			
		*F*_6,216_	*p*	$\eta_{p}^{2}$	*F*_1,36_	*p*	$\eta_{p}^{2}$
ROI 1: L amygdala, anterior insula, inferior temporal gyrus, anterior division, inferior frontal gyrus, frontal operculum, frontal pole	3110	1.86	0.089	0.049	0.296	0.590	0.01
ROI 2: L lateral occipital fusiform cortex, lateral occipital cortex inferior division, occipital fusiform gyrus	4542	3.92	***0.001***	0.10	5.15^a^	*0.029*^a^	0.13^a^
ROI 3: R amygdala, anterior insula, inferior temporal gyrus, anterior division, inferior frontal gyrus, frontal operculum, frontal pole	3090	2.42	*0.040*	0.06	1.97	0.169	0.05
ROI 4: R lateral occipital fusiform cortex, lateral occipital cortex inferior division, occipital fusiform gyrus	4806	5.26	***<0.0005***	0.13	1.57	0.218	0.04
ROI 5: Dorsal anterior cingulate cortex, paracingulate cortex, superior frontal gyrus, juxtapositional lobule cortex	5044	2.83	***0.011***	0.07	1.40	0.244	0.04
ROI 6: L and R thalamus	1125	0.96	0.451	0.03	NT	NT	NT
ROI 7: L Occipital pole	113	2.27	0.052	0.06	0.03	0.870	0.00
ROI 8: L intracalcarine cortex	133	6.11	***<0.0005***	0.15	0.12	0.734	0.00
ROI 9: posterior cingulate cortex	122	0.84	0.540	0.02	NT	NT	NT
ROI 10: L supramarginal gyrus	397	3.23	***0.005***	0.08	0.85	0.361	0.02
ROI 11: R precentral gyrus	215	0.37	0.900	0.01	NT	NT	NT
ROI 12: precuneus	301	2.26	*0.039*	0.06	3.88^a^	0.056^a^	0.10^a^


*All significant values (*p* < 0.05) are in italics, and those that reach FDR-corrected significance are in bold (corrected for 12 ROIs). NT, not tested: areas without significant main effect of morph are not tested for group effects.^a^Group effect caused by more classifications as spider in the low spider fear group. L = left; R = right.*

It is noteworthy that the classification did not change substantially when we corrected for average activation, which we did by subtracting the signal per trial, averaged across voxels within that ROI (i.e., preserving the spatial pattern, but scaling the activation so that every trial’s mean activation in a particular ROI was zero).

## Discussion

The aim of the present study was to examine how fear influences the processing of ambiguous stimuli. We used SVM classification to explore whether overgeneralization of fear is associated with functional anomalies in regions traditionally associated with early perception and object identification, and/or with regions involved in saliency and higher cognitive processes. Identifying where in the information-processing stream fear affects ambiguity resolution could potentially indicate whether this bias is primarily perceptual, or conceptual in nature.

In line with previous findings (Kolassa et al., 2007), individuals with HSF were more likely to classify ambiguous morphs as spiders than individuals with LSF. Unexpectedly, neither average activation, nor SVM classification in 12 functional ROIs revealed a pattern that mirrored the behavioral effect. We even obtained some evidence that response patterns in visual association areas related to ambiguous stimuli were more likely to be classified as spiders when individuals were *not* afraid of spiders.

These findings seem to point to a methodological limitation of the current paradigm. Unlike most studies, which used either pictures of phobogenic material (Dilger et al., 2003) or schematic morphs (Kolassa et al., 2007), we alternately presented morphs and pictures of real spiders. In the SVM classification, we trained a classifier on the unambiguous pictures to classify the schematic morphs. This seemed the most ecologically valid approach, as we were interested in the degree to which the morphs would resemble ‘true’ spiders or flowers, not in the similarity between schematic morphs *per se*. However, by mixing the two types of stimuli, we may have unintentionally deflated the valence of the spider drawings, skewing the classification of these stimuli. Although these morphs could have been a valid representation of the fearful category if presented alone, the (anticipated) presence of stimuli that are substantially more arousing may have turned the morphs into relatively safe stimuli. For individuals without spider fear both classes of unambiguous stimuli were virtually neutral, so training a classifier on these stimuli showed a linear increase in the likelihood that an ambiguous morph is classified as a spider. In contrast, for individuals with HSF the classifier is trained on two very distinct categories: one neutral and one highly emotional. This is also evident from the fact that these unambiguous categories are better classified – thus more distinct – in high fearful individuals than in low fearful individuals. In this scenario, a relatively neutral morph is classified as a flower, probably not on the basis of perceptual or even conceptual similarity, but on the absence of a strong emotional response. Between-run classification, with ambiguous and unambiguous stimuli presented in separate runs, could partially solve this problem: without the continuous anticipation of the (terrifying) unambiguous spider pictures, the spider drawings could be semantically categorized as spiders, instead of merely being categorized as ‘relatively safe.’ Between-run classification also has the advantage of ensuring complete independence between train and test set, thereby preventing possible biases in pattern classification (Mumford et al., 2014). Yet, even in separate runs, the unambiguous spider pictures will undoubtedly elicit a much stronger emotional response than the morphs, which may still hamper a balanced classification of stimulus categories if the unambiguous stimuli were to be used for training. This exemplifies that it is methodologically challenging to classify emotional and neutral stimuli in groups that differ in their judgment of emotionality.

Even though the design may have skewed the classification of morphs in some areas of the brain, participants did eventually indicate that they had seen a spider. Given that every behavioral response must have a neural correlate, one would assume that it should be possible with SVM classification to detect an area that codes for this behavior. We did not find such an area. Again, this suggests that our study design may not have been optimal for detecting subtle effects. While overgeneralization of fear was observed at the level of the group, individual behavior consisted of three discrete responses per stimulus type, yielding a proportion of stimuli classified as flower or spider. Thus, individual behavior was not necessarily representative of the group average. Although we based our behavioral task on previous research (Kolassa et al., 2007), future research should explore the possibility of having participants evaluate ambiguous stimuli on a continuous scale, which seems a more sensitive approach for linking individual behavior to brain data. Further, it may be useful in future research to add a jitter between the presentation of a stimulus and presentation of the response screen, so that responses could be independently modeled as regressor of no interest. This would make it easier to separate decision-making processes from basic perceptual processes. Lastly, the number and spacing of stimuli used in this experiment were based on previous studies. In these studies pattern analysis was applied to distinguish face and house stimuli (Epstein and Kanwisher, 1998; Haxby et al., 2001) and to assess the effects of Pavlovian conditioning (Visser et al., 2011, 2013, 2015, 2016), which is a powerful manipulation. The present manipulation – gradually morphing a flower into a spider – was presumably subtler and therefore required a greater number of trials. Increasing the power and using different behavioral measures would likely yield stronger effects and would open up avenues for model-based searchlights, using (continuous) representational similarity analysis (Kriegeskorte et al., 2008; Kriegeskorte, 2011) instead of the rather coarse (dichotomous) classification analysis. Approaches like these could further elucidate how fear influences the perception, categorization and interpretation of ambiguous stimuli.

In sum, while we found behavioral evidence for over-generalization of fear in spider phobia, replicating previous findings (Kolassa et al., 2007), we were not able to identify a neural signature of this bias. The question as to where in the information-processing stream this bias emerges therefore remains a topic for further investigation.

## Author Contributions

RV, PH, and MK designed the research; RV and PH performed the research and analyzed the data; HS and RZ contributed analytic tools; RV and MK wrote the manuscript.

## Conflict of Interest Statement

The authors declare that the research was conducted in the absence of any commercial or financial relationships that could be construed as a potential conflict of interest.

## Footnotes

1 www.fmrib.ox.ac.uk/fsl
